# Anti-CD20 therapy depletes activated myelin-specific CD8^+^ T cells in multiple sclerosis

**DOI:** 10.1073/pnas.1915309116

**Published:** 2019-11-20

**Authors:** Joseph J. Sabatino, Michael R. Wilson, Peter A. Calabresi, Stephen L. Hauser, Jonathan P. Schneck, Scott S. Zamvil

**Affiliations:** ^a^Department of Neurology, University of California, San Francisco, CA 94158;; ^b^Weill Institute for Neurosciences, University of California, San Francisco, CA 94158;; ^c^Department of Neurology, Johns Hopkins School of Medicine, Baltimore, MD 21205;; ^d^Department of Pathology, Johns Hopkins School of Medicine, Baltimore, MD 21287;; ^e^Program in Immunology, University of California, San Francisco, CA 94143

**Keywords:** multiple sclerosis, CD8^+^ T cells, myelin antigen, anti-CD20 therapy

## Abstract

CD8^+^ T cells are believed to play an important role in multiple sclerosis (MS), yet their role in MS pathogenesis remains poorly defined. Although myelin proteins are considered potential autoantigenic targets, prior studies of myelin-reactive CD8^+^ T cells in MS have relied on in vitro stimulation, thereby limiting accurate measurement of their ex vivo precursor frequencies and phenotypes. Peptide:MHC I tetramers were used to identify and validate 5 myelin CD8^+^ T cell epitopes, including 2 newly described determinants in humans. The validated tetramers were used to measure the ex vivo precursor frequencies and phenotypes of myelin-specific CD8^+^ T cells in the peripheral blood of untreated MS patients and HLA allele-matched healthy controls. In parallel, CD8^+^ T cell responses against immunodominant influenza epitopes were also measured. There were no differences in ex vivo frequencies of tetramer-positive myelin-specific CD8^+^ T cells between MS patients and control subjects. An increased proportion of myelin-specific CD8^+^ T cells in MS patients exhibited a memory phenotype and expressed CD20 compared to control subjects, while there were no phenotypic differences observed among influenza-specific CD8^+^ T cells. Longitudinal assessments were also measured in a subset of MS patients subsequently treated with anti-CD20 monoclonal antibody therapy. The proportion of memory and CD20^+^ CD8^+^ T cells specific for certain myelin but not influenza epitopes was significantly reduced following anti-CD20 treatment. This study, representing a characterization of unmanipulated myelin-reactive CD8^+^ T cells in MS, indicates these cells may be attractive targets in MS therapy.

## Significance

Multiple sclerosis (MS) is an inflammatory demyelinating disease of the central nervous system. CD8^+^ T cells have been strongly implicated in MS pathogenesis, but it is unclear whether myelin is a CD8^+^ T cell autoantigenic target in MS. This study demonstrated that while myelin-specific CD8^+^ T cells are present at similar frequencies in untreated MS patients and healthy subjects, the proportion of memory and CD20-expressing myelin-specific CD8^+^ T cells was increased in MS patients, suggesting prior antigen encounter. This activated phenotype was reversible as the memory and CD20-expressing populations of certain myelin-specific CD8^+^ T cells were reduced following anti-CD20 treatment.

The adaptive immune system, including CD4^+^ T cells and B cells, has long been implicated in the pathogenesis of multiple sclerosis (MS) (1, 2), a chronic inflammatory demyelinating disease of the central nervous system (CNS). Although CD8^+^ T cells are primarily recognized for their cytotoxic function in antiviral and antitumor immunity, compelling evidence suggests they have an important role in MS. CD8^+^ T cells are enriched in the perivascular space and leading edge of MS white matter lesions (3–7), where they greatly outnumber CD4^+^ T cells (4, 6, 8, 9) and are also present in the majority of cortical lesions (10). In contrast to CD4^+^ T cells, CD8^+^ T cells in MS lesions are clonally expanded (11–13), suggesting antigen-driven proliferation. In individual MS patients, common clonal CD8^+^ T cell populations are present within the lesions and normal-appearing white matter (11, 14). Several MHC I alleles, including HLA-A*02:01 (HLA-A2) and HLA-A*03:01 (HLA-A3), are known to alter MS susceptibility (15, 16). MHC I is specifically up-regulated in microglia, macrophages, astrocytes, and oligodendrocytes of acute MS lesions (17). In addition, CD8^+^ T cells in MS lesions are found in direct contact with oligodendrocytes (8), suggesting CD8^+^ T cells may contribute directly to demyelinating pathology.

Myelin is considered a putative target autoantigen in MS (18), and myelin-specific CD8^+^ T cells are well known to contribute to CNS pathology in experimental autoimmune encephalomyelitis (EAE) (19–23). A number of studies have described the reactivity of human CD8^+^ T cells against myelin antigens (24–30); however, reports are conflicting whether myelin-specific CD8^+^ T cell responses differ between MS patients and controls (24, 27–30). Much of the prior work was limited by a reliance on functional assays, precluding the sensitive detection of unmanipulated epitope-specific CD8^+^ T cell populations.

To overcome these limitations, we generated a large panel of myelin peptide:MHC I (pMHC I) tetramers, considered the gold standard for detection of antigen-specific CD8^+^ T cells (31). We sought to identify and validate myelin-specific CD8^+^ T cell epitopes restricted by HLA-A2 and HLA-A3 alleles by combining pMHC I tetramer detection and functional reactivity to cognate myelin antigen. Using this approach, we validated 5 myelin CD8^+^ T cell epitopes, including 2 epitopes that have not been previously reported in humans. Combinatorial tetramer staining (32, 33) and enrichment (34) strategies were employed to identify myelin-specific CD8^+^ T cells ex vivo from the peripheral blood. In comparison, we also measured CD8^+^ T cell responses to immunodominant epitopes of influenza, a widely encountered foreign antigen. We observed that the frequencies of myelin-specific CD8^+^ T cells did not differ between MS patients and control subjects for any of the myelin epitopes. We also assessed the phenotypes of these CD8^+^ T cell populations by the expression of memory markers as well as CD20, which is increased on memory CD8^+^ T cells in MS patients and reduced following anti-CD20 monoclonal antibody (mAb) treatment (35, 36). Interestingly, memory myelin-specific CD8^+^ T cells were increased in MS patients compared to controls, indicating prior exposure to antigen. The proportion of myelin-specific CD8^+^ T cells expressing CD20 was also increased in MS patients, consistent with an increased activation state. No such phenotypic differences were observed between influenza-specific CD8^+^ T cells. Myelin-specific CD8^+^ T cells with the highest memory phenotype and CD20 expression at baseline had a significant reduction in these markers following anti-CD20 mAb treatment. These studies suggest that myelin-specific CD8^+^ T cells have an activated phenotype in MS patients, thereby permitting their preferential depletion by anti-CD20 mAb therapy.

## Results

### Identification of Myelin CD8^+^ T Cell Epitopes.

A tetramer-based expansion protocol, previously described for the identification of tumor neo-antigen–specific CD8^+^ T cells (37), was employed for screening candidate myelin CD8^+^ T cell epitopes (*Materials and Methods*). Screening for HLA-A2– and HLA-A3–restricted myelin T cell epitopes was focused on 5 key myelin proteins expressed in the CNS: proteolipid protein (PLP), myelin basic protein (MBP), myelin oligodendrocyte glycoprotein (MOG), myelin-associated glycoprotein (MAG), and 2′,3′-cyclic-nucleotide 3′-phosphodiesterase (CNP). Candidate myelin epitopes were selected by *in silico* epitope prediction for HLA-A2 and HLA-A3 (*SI Appendix*, Table S1), followed by measurement of relative binding of individual myelin peptides to each MHC I protein (*SI Appendix*, Fig. S1).

**Fig. 1.**
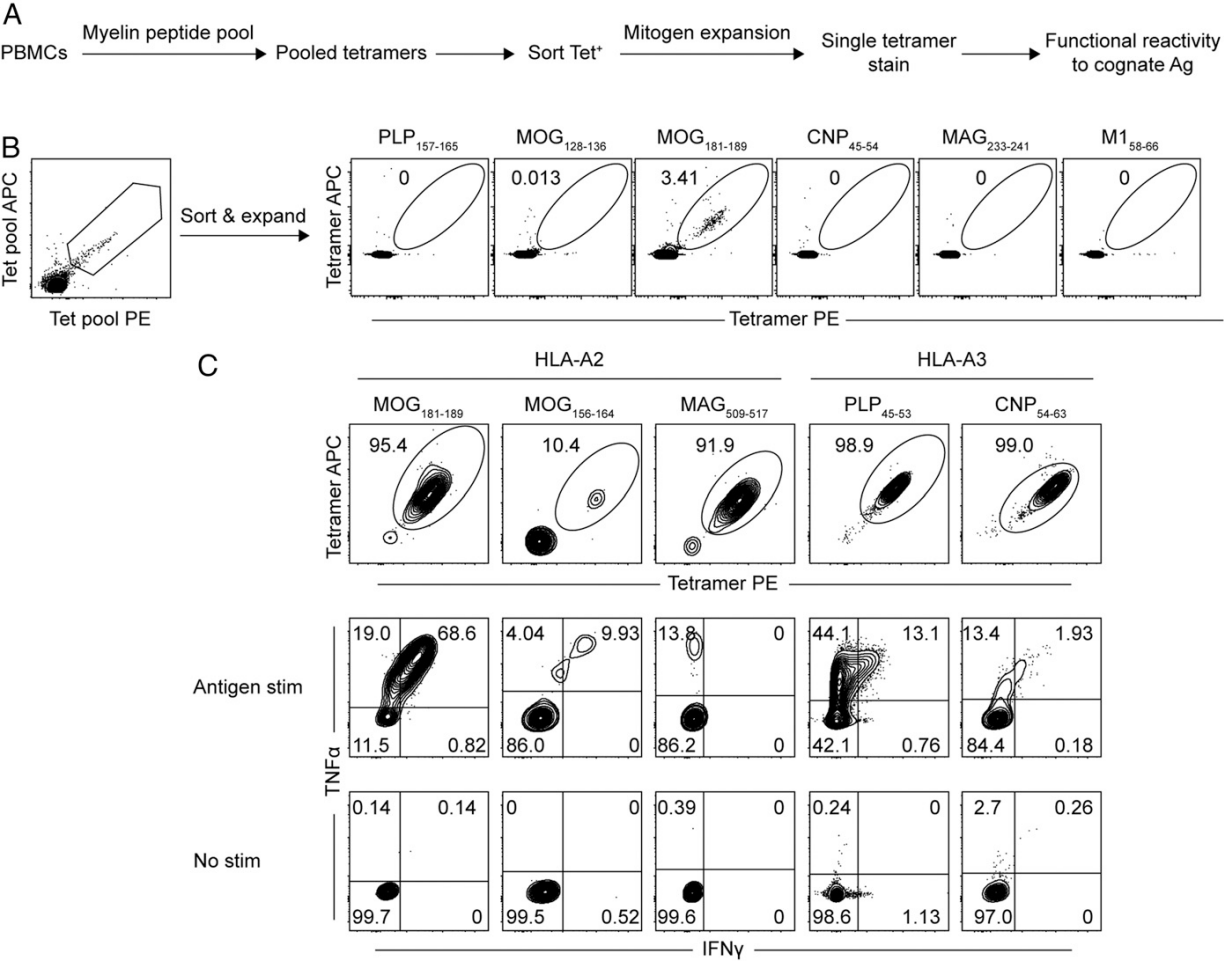
Validation of myelin-specific CD8^+^ T cell epitopes. Overview of screening and confirmation of myelin-specific CD8^+^ T cells starting from pools of myelin peptides (*A*). In this approach, PBMCs from a cohort of HLA-A2^+^ and HLA-A3^+^ untreated RR-MS patients were stimulated in vitro with pools of myelin peptides (see Table 1 for tested peptides) for 1 wk. After staining with pools of cognate myelin pMHC I tetramers, tetramer-positive CD8^+^ T cells were sorted and expanded with mitogen for 2 to 4 wk. Expanded cells were tested for specific binding to individual tetramers. Myelin antigens demonstrating tetramer binding were then tested for functional reactivity to cognate antigen by cytokine production. Only those myelin epitopes demonstrating both specific tetramer binding and functional reactivity were considered validated. A representative experiment (*B*) demonstrating identification of MOG_181–189_:HLA-A2 tetramer-positive CD8^+^ T cells. The HLA-A2–restricted influenza antigen M1_58–66_ was used as an additional negative control to confirm specificity of MOG_181–189_ tetramer binding. The 5 myelin epitopes that were validated by tetramer binding and intracellular cytokine production to cognate antigen are shown (*C*).

Pools of selected myelin peptides were used to stimulate peripheral blood mononuclear cells (PBMCs) from a cohort of untreated HLA-A2^+^ and HLA-A3^+^ relapsing–remitting MS (RR-MS) patients. Pooled myelin tetramer-positive CD8^+^ T cells were sorted, expanded with mitogen, and screened for their ability to bind each myelin pMHC I tetramer individually (Fig. 1*A*). As shown in one representative experiment (Fig. 1*B*), expanded CD8^+^ T cells only bound to HLA-A2–restricted MOG_181–189_ tetramer. There was no binding to the immunodominant HLA-A2–restricted influenza A epitope M1_58–66_, supporting specificity of MOG_181–189_ tetramer binding. Functional reactivity and HLA restriction of MOG_181–189_ tetramer-positive CD8^+^ T cells was demonstrated by intracellular cytokine production following stimulation with cognate antigen-loaded T2 cells (see *Materials and Methods* for further details) (Fig. 1*C*). Using this stepwise approach, 3 HLA-A2 (MOG_181–189_, MOG_156–164_, and MAG_509–517_) and 2 HLA-A3 (PLP_45–53_ and CNP_54–63_) CD8^+^ T cell epitopes were validated (Fig. 1*C*). The determinants MOG_156–164_:HLA-A2, MAG_509–517_:HLA-A2, and PLP_45–53_:HLA-A3 have previously been described in humans (24, 25, 30). To our knowledge, MOG_181–189_:HLA-A2 and CNP_54–63_:HLA-A3 have not been described in humans. Of interest, MOG_181–189_ was previously reported as a CD8^+^ T cell epitope in HLA-A2 transgenic mice (21), and both the MOG_181–189_ and PLP_45–53_ epitopes were pathogenic in humanized mouse models of EAE (21, 22). A potential pathogenic role for these populations is supported by the finding that all of the isolated myelin-specific CD8^+^ T cell populations produced significant amounts of IFNγ, TNFα, IL-2, GM-CSF, and CD107a (*SI Appendix*, Fig. S2 *A* and *B*).

### Ex Vivo Frequency Analysis of Myelin CD8^+^ T Cells.

**Fig. 2.**
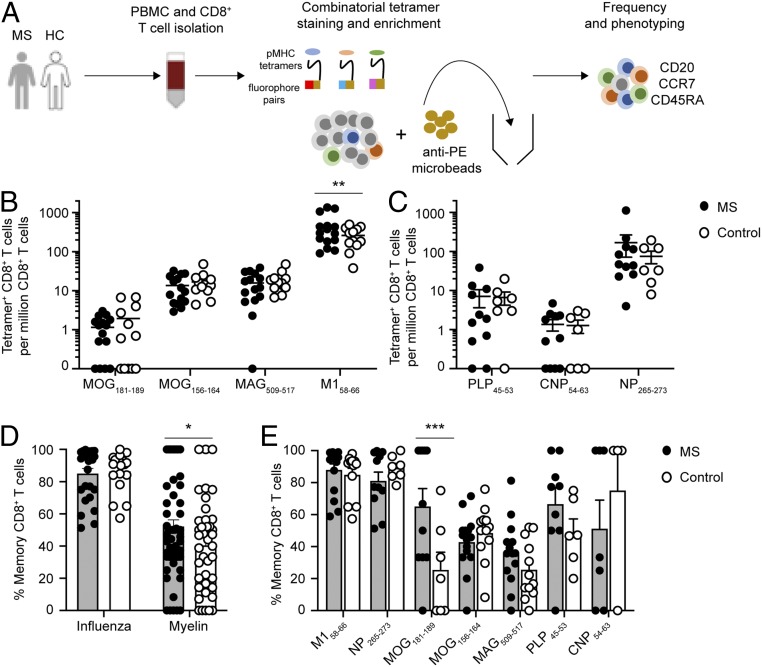
Ex vivo characterization of myelin-specific CD8^+^ T cells in the peripheral blood. Depiction of the experimental paradigm for ex vivo characterization of tetramer-positive CD8^+^ T cells (*A*). PBMCs were collected from a cohort of untreated RR-MS patients and HLA allele-matched healthy controls. Purified CD8^+^ T cells were stained with a panel of tetramers with unique combinations of dual fluorophores containing at least one PE fluorophore, allowing enrichment with anti-PE microbeads over a magnetic column. The frequencies and cell surface marker expression of each antigen-specific CD8^+^ T cell population were then determined. The ex vivo frequencies of HLA-A2–restricted (*B*) and HLA-A3–restricted (*C*) myelin- and influenza-specific CD8^+^ T cells in MS patients (*n* = 15 HLA-A2; *n* = 11 HLA-A3) and control subjects (*n* = 12 HLA-A2; *n* = 7 HLA-A3) were compared (note: 2 of the MS patients and 2 of the control subjects were HLA-A2^+^/A3^+^, thus accounting for the increased number of data points relative to the number of individual subjects listed in Table 1). The percentages of pooled (*D*) or individual (*E*) memory influenza- and myelin-specific CD8^+^ T cells were compared between MS patients (*n* = 26 samples) and HLA allele-matched controls (*n* = 19 samples). Memory status was defined by pooling all memory phenotypes (combined central memory CCR7^+^ CD45RA^−^, effector memory CCR7^−^CD45RA^−^, and TEMRA CCR7^−^ CD45RA^+^) in order to increase the number of cells for analysis). The circles represent individual samples (filled circles, MS; open circles, control). For *B* and *C*, the line represents the mean ± SEM; for *D* and *E*, the top of the bar graph represents the mean ± SEM. Only samples with detectable tetramer-positive CD8^+^ T cells were included for analysis. A 2-way ANOVA with multiple comparisons was used for comparisons between MS patients and control subjects (*P < 0.05, **P = 0.002, and ***P = 0.001).

The circulating frequencies of autoreactive T cells in the peripheral blood are rare (∼1 in 10^5^ to 10^6^ T cells) (38). We employed a combinatorial tetramer staining (32, 33) and magnetic bead enrichment strategy (31, 34, 39) (Fig. 2*A*) in order to measure multiple antigen-specific T cells ex vivo in an accurate and sensitive manner. We measured the ex vivo frequencies of the 5 validated myelin CD8^+^ T cell determinants in the peripheral blood of a second cohort of untreated RR-MS patients and HLA allele-matched healthy controls (Table 1). The immunodominant influenza epitopes M1_58–66_ (HLA-A2) and NP_265–273_ (HLA-A3) were used as comparisons to ubiquitous viral-specific CD8^+^ T cells. Antigen-specific CD8^+^ T cells were identified from a single PBMC sample using tetramer pools containing unique combinations of dual fluorophores (*SI Appendix*, Table S2). Myelin-specific CD8^+^ T cells were readily detectable in nearly all individuals. Strikingly, there were no significant differences in the frequencies of any of the myelin-specific CD8^+^ T cell populations in MS patients in comparison to HLA allele-matched healthy controls (Fig. 2 *B* and *C*). Influenza-specific CD8^+^ T cells were detected at frequencies 1 to 2 orders of magnitude higher than those specific for myelin antigen (mean, 3.73 × 10^−4^ ± 0.65 and 1.33 × 10^−4^ ± 0.60 for HLA-A2 and HLA-A3, respectively). Interestingly, influenza-specific (M1_58–66_) CD8^+^ T cells in HLA-A2^+^ RR-MS patients were significantly increased compared to HLA-allele matched healthy controls, but no significant difference was observed in influenza-specific CD8^+^ T cells (NP_265–273_) in HLA-A3^+^ individuals (Fig. 2 *B* and *C*).

**Table 1.** Study subject characteristics

Subjects	*N*	Age	Female	HLA-A2^+^/A3^+^	Active relapse	Treatment naive	Anti-CD20 mAb at T2
RR-MS	24	38 (18–54)	17 (71%)	15/11	10 (42%)	22 (92%)	9 (38%)
Control	17	31 (23–57)	10 (59%)	12/7	N/A	N/A	N/A

The number (*N*), mean age (and range), and percent female for RR-MS patients and control subjects used for ex vivo CD8^+^ T cell frequency and phenotype analysis are shown. The number of HLA-A2^+^ and HLA-A3^+^ individuals (some codominantly expressed both MHC I alleles) for each group is shown. The number (and percent) of RR-MS patients experiencing an active relapse within the past 30 d (confirmed by contrast-enhancing lesion on MRI) and the percent who were naive to DMT at the time of their baseline sample acquisition are also shown. The number (and percent) of RR-MS patients who were subsequently treated with anti-CD20 mAb and underwent repeat analysis at follow-up time point 2 (T2) is shown.

### Memory Status of Myelin-Specific CD8^+^ T Cells.

The differentiation states of ex vivo antigen-specific CD8^+^ T cells were explored further by evaluating their CCR7 and CD45RA expression, prototypic markers of naive and memory status (*SI Appendix*, Fig. S4*A*). In comparing MS patients to control subjects, there was a significant reduction in naive (28.1 ± 2.8% vs. 36.7 ± 3.4%, respectively) and a corresponding increase in effector memory (41.2 ± 3.5% vs. 26.6 ± 2.4%, respectively) fractions in total CD8^+^ T cells (i.e., irrespective of antigen specificity) in MS patients compared to control subjects (*SI Appendix*, Fig. S4*B*), consistent with prior reports (40, 41). Because of the limited numbers of antigen-specific T cells available for phenotyping, we compared the memory status of all tetramer-positive CD8^+^ T cells by naive (CCR7^+^ CD45RA^+^) or memory (combined central memory CCR7^+^ CD45RA^−^, effector memory CCR7^−^CD45RA^−^, and TEMRA CCR7^−^ CD45RA^+^). Influenza-specific CD8^+^ T cells exhibited a predominantly memory phenotype, and there were no differences in memory status between MS patients and control subjects (Fig. 2 *D* and *E*). As a whole, the memory status of all combined myelin-specific CD8^+^ T cells in MS patients (52.2 ± 4.1%) was significantly increased compared to controls (40.5 ± 4.4%) (Fig. 2*D*). In particular, memory MOG_181–189_-specific CD8^+^ T cells were significantly increased in MS patients (65.1 ± 11.1%) compared to controls (25.5 ± 11.0%) (Fig. 2*E*). CD8^+^ T cells responding to 2 additional myelin determinants, MAG_509–517_ and PLP_45–53_, showed nonsignificant increases in memory status in MS patients (Fig. 2*E*). These data suggest that although the frequencies of myelin-specific CD8^+^ T cells do not differ between MS patients and control individuals, those T cells from MS patients are more likely to exhibit a memory phenotype.

### CD20 Expression of Myelin-Specific CD8^+^ T Cells.

**Fig. 3.**
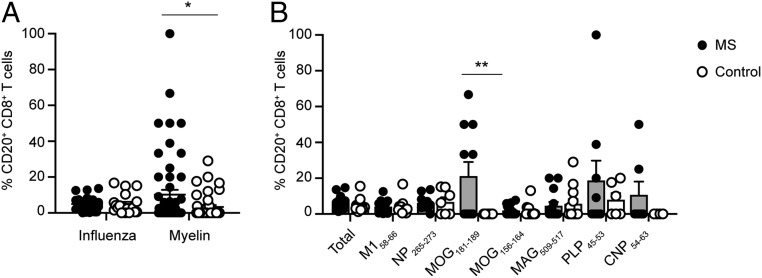
CD20 expression of antigen-specific CD8^+^ T cells. The overall frequencies of total influenza- and myelin-specific CD20^+^ CD8^+^ T cells were compared between MS patients (*n* = 26 samples) and control subjects (*n* = 19 samples) (*A*). The percentage of CD20-expressing CD8^+^ T cells was compared for all CD8^+^ T cells as well as for each individual epitope between MS patients and controls (*B*). The circles represent individual samples (filled circles, MS; open circles, control), the line represents the mean ± SEM in *A*, and the top of the bar graph represents the mean ± SEM in *B*. Only samples with detectable tetramer-positive CD8^+^ T cells were included for analysis. A 2-way ANOVA with multiple comparisons was used for comparisons between MS patients and control subjects (*P = 0.01; **P = 0.0002).

Although typically associated with B cells, CD20 is also expressed on a subset of highly activated proinflammatory T cells and is typically found in memory T cell populations (35, 36, 42). CD20^+^ T cells are reported to be increased in MS patients (35, 36), are present in MS lesions (43), and are reduced by anti-CD20 mAb therapies (35, 42, 44). The expression of CD20 was therefore measured on ex vivo antigen-specific CD8^+^ T cells (*SI Appendix*, Fig. S5*A*). Interestingly, the frequencies of pooled myelin-specific CD20^+^ CD8^+^ T cells was significantly higher in MS patients (10.1 ± 2.7%) compared to control subjects (3.3 ± 1.1%), whereas there were no differences in influenza-specific CD20^+^ CD8^+^ T cells (5.0 ± 0.7% and 4.9 ± 1.2%, respectively) (Fig. 3*A*). The percentage of MOG_181–189_:HLA-A2 CD8^+^ T cells expressing CD20 was significantly increased in MS patients (21.2 ± 7.8%) compared to HLA allele-matched controls (0.0 ± 0.0%) (Fig. 3*B*). There was also an increase in PLP_45–53_:HLA-A3 CD20^+^ CD8^+^ T cells in MS patients compared to controls that was nonsignificant (18.7 ± 11.1% and 7.9 ± 3.8%, respectively; *P* = 0.08). This represented a substantial enrichment in CD20 expression in comparison to the total frequency of CD20^+^ CD8^+^ T cells (5.5 ± 0.7% in MS patients; 4.4 ± 0.8% in controls). Influenza-specific CD20^+^ CD8^+^ T cell populations exhibited a predominantly memory phenotype, consistent with the known activated state of CD20^+^ T cells (35, 36). The memory status of CD20-expressing myelin-specific CD8^+^ T cells was variable, but with an overall significant increase in myelin-specific memory CD20^+^ CD8^+^ T cells in MS patients (53.7 ± 10.3%) compared to control subjects (27.0 ± 9.7%) (*SI Appendix*, Fig. S5*B*). Thus, these findings provide further support that a greater proportion of myelin-specific CD8^+^ T cells in MS patients have encountered antigen compared to control subjects.

### Effects of Anti-CD20 Treatment on Myelin-Specific CD8^+^ T Cells.

**Fig. 4.**
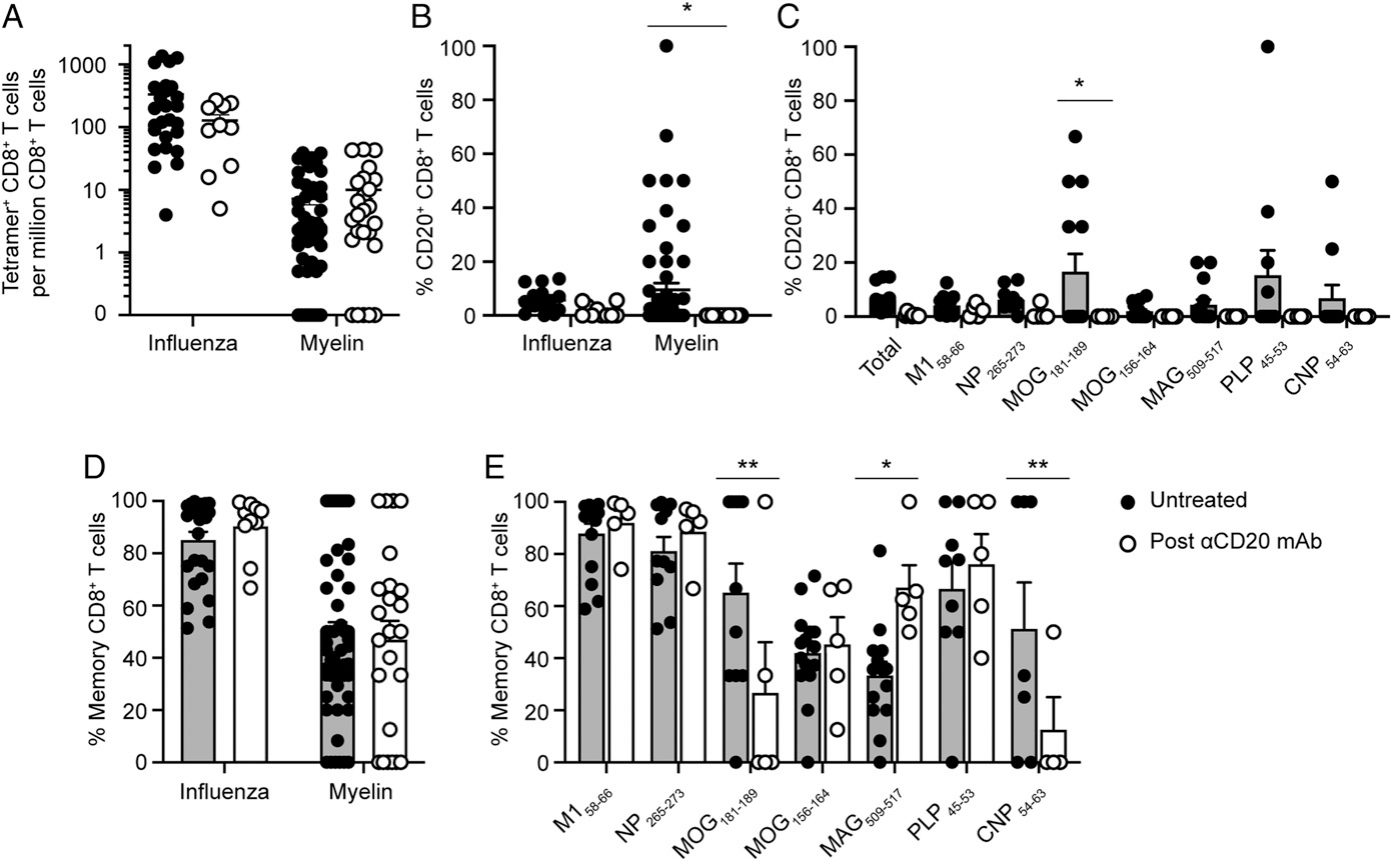
Effects of anti-CD20 mAb treatment on antigen-specific CD8^+^ T cells. The frequencies of pooled influenza- and myelin-specific CD8^+^ T cells was compared for untreated MS patients (*n* = 26 samples) and a subset of the same patient cohort subsequently treated with anti-CD20 mAb (*n* = 10 samples) (*A*). The mean percentage of CD20^+^ CD8^+^ T cells specific for pooled influenza and myelin epitopes (*B*) or each individual epitope (*C*) was compared between untreated MS patients and following anti-CD20 mAb treatment. The mean percentage of memory CD8^+^ T cells for pooled influenza and myelin epitopes (*D*) or each individual epitope (*E*) before and after anti-CD20 mAb treatment is also shown. The circles represent individual samples (filled circles, untreated; open circles, post-αCD20 mAb). For *A* and *B*, the line represents the mean ± SEM; for *C*–*E*, the top of the bar graph represents the mean ± SEM. Only samples with detectable tetramer-positive CD8^+^ T cells were included for analysis in *B*–*E*. A mixed-effects model for repeated measures with multiple comparisons was used for comparisons of untreated and anti-CD20 mAb-treated samples (*P < 0.05; **P ≤ 0.01).

Anti-CD20 mAb therapies, including rituximab and ocrelizumab, have become a mainstay of MS treatment due to their high efficacy (45, 46). Since CD20 expression is increased in myelin-specific CD8^+^ T cells in MS patients, we therefore asked whether these T cells may be preferentially depleted following anti-CD20 mAb treatment. The effect of anti-CD20 mAb was examined by comparing MS patients before (i.e., untreated) and after anti-CD20 mAb treatment (*SI Appendix*, Fig. S6*A*). There was no significant effect on the overall (Fig. 4*A*) or individual (*SI Appendix*, Fig. S6 *B* and *C*) frequencies of influenza- and myelin-specific CD8^+^ T cells following anti-CD20 mAb treatment, with the exception of a small reduction in M1_58–66_-specific CD8^+^ T cells (*SI Appendix*, Fig. S6*B*). There was a significant reduction in the frequency of pooled myelin-specific, but not influenza-specific CD20^+^ CD8^+^ T cells following anti-CD20 mAb treatment (Fig. 4*B*). Anti-CD20 mAb treatment was associated with significant reductions in the percentages of MOG_181–189_:HLA-A2 CD20^+^ CD8^+^ T cells and nonsignificant reductions for PLP_45–53_:HLA-A3 (*P* = 0.11) and CNP_54–63_:HLA-A3 (*P* = 0.14) (Fig. 4*C*). There was no significant decrease in the percentages of total influenza- and myelin-specific memory CD8^+^ T cells (Fig. 4*D*). Anti-CD20 mAb treatment was associated with significant reductions in the percentage of memory CD8^+^ T cells specific for MOG_181–189_:HLA-A2 and CNP_54–63_:HLA-A3 with an increase in MAG_509–517_:HLA-A2 memory CD8^+^ T cells (Fig. 4*E*).

## Discussion

Compelling evidence indicates that CD8^+^ T cells play an important role in MS. CD8^+^ T cells are abundant and clonally expanded in MS lesions (3–7), and certain MHC I alleles are linked with MS susceptibility (15, 16). Indeed, it was recently shown that clonally expanded CD8^+^ T cells are an early feature in the CSF of MS-discordant monozygotic twins with subclinical neuroinflammation (47). CD8^+^ T cells also are reduced by a number of MS disease-modifying therapies (DMTs), including S1P receptor modulators, which are correlated with reductions in biomarkers of CNS injury (48). CD8^+^ T cells specific for myelin antigens are also pathogenic in various EAE models (19–23). Prior efforts to study myelin-specific CD8^+^ T cells have been hampered by technical limitations and reliance on in vitro manipulation (24–30). In this study, we employed pMHC I tetramer-based methods to unambiguously identify myelin-specific CD8^+^ T cell populations directly from the peripheral blood without in vitro stimulation or manipulation. In this study, we identified 2 myelin determinants not previously described in humans, MOG_181–189_:HLA-A2 and CNP_54–63_:HLA-A3, as well as several previously reported myelin-specific CD8^+^ T cell epitopes (21, 24, 25, 30). By using a highly sensitive and specific combinatorial tetramer staining and enrichment strategy, we showed that the ex vivo frequencies of myelin-specific CD8^+^ T cells in the peripheral blood did not differ between MS patients and MHC I allele-matched control subjects. These findings are consistent with reports that self-reactive CD8^+^ T cells are present at similar frequencies in individuals with and without autoimmune disease (39, 49) and reinforce the principle that central tolerance does not completely eliminate all self-reactive T cells. Despite the lack of quantitative differences, we found an increased proportion of memory myelin-specific CD8^+^ T cells in MS patients compared to control subjects, indicating prior activation by antigen. In vitro expansion of these myelin-specific CD8^+^ T cells revealed the production of proinflammatory cytokines. Two of the epitopes we studied, MOG_181–189_:HLA-A2 and PLP_45–53_:HLA-A3, are pathogenic in humanized HLA transgenic mouse models of EAE (21, 22). In addition, myelin-reactive human T cells have the capacity to induce CNS inflammation in immunodeficient mice (50). These findings therefore support the possibility that myelin-CD8^+^ T cells may contribute to MS pathogenesis.

Although CD20 is a hallmark cell surface molecule expressed by B cells and is the target for B cell-depleting therapy in MS, it is now recognized that some T cells express CD20, which is expressed by a higher proportion of CD8^+^ T cells compared to CD4^+^ T cells (35, 36, 42, 51). CD8^+^ T cells expressing CD20 have been previously demonstrated to be highly activated proinflammatory cytokine-producing memory T cells bearing CNS-homing chemokine receptors and adhesion molecules (36, 42), thus highlighting their pathogenic potential. In addition, CD20^+^ T cells have been directly implicated in MS pathogenesis (35, 36, 43). The precise mechanism(s) responsible for the benefit of anti-CD20 mAb in MS is not entirely clear and may include effects on T cells (52, 53). The finding that the frequency of CD20-expressing myelin-specific CD8^+^ T cells is increased in MS patients compared to control individuals is consistent with an increased activation state and suggested that these cells could be targeted by anti-CD20 mAb therapy. Indeed, our findings confirm that CD20-expressing myelin-specific CD8^+^ T cells are preferentially depleted by anti-CD20 mAb treatment. Similarly, the proportion of certain memory myelin-specific CD8^+^ T cells was significantly reduced following anti-CD20 mAb therapy, consistent with the memory status of CD20^+^ CD8^+^ T cells. Surprisingly, the CD20 expression of influenza-specific CD8^+^ T cells was reduced to a lesser degree by anti-CD20 mAb. The reason for this discrepancy is not clear; however, it is important to note that the molecular mechanisms by which T cells express CD20 have not been fully elucidated, and CD20^+^ T cell reconstitution occurs earlier (3 to 6 mo) than for B cells (>6 mo) following anti-CD20 mAb treatment (35, 42). Most of the patients in the longitudinal arm of this study were treated with anti-CD20 mAb 3 to 6 mo prior; therefore, it is possible that CD20^+^ CD8^+^ T cells specific for influenza reconstituted more quickly than those specific for myelin antigen.

Our study demonstrated an increase in memory myelin-specific CD8^+^ T cells in MS patients. Given the inherent promiscuity of the T cell receptor (54, 55), it is possible that the initial priming of those T cells could be due to a cross-reactive antigen (e.g., viral antigen). Nonetheless, these findings point to increased antigen experience of CD8^+^ T cells capable of responding to myelin antigens in MS patients compared to healthy controls. The underlying cause for the increase in myelin-specific CD8^+^ T cell activation in MS patients remains unclear. Although HLA-A2 and HLA-A3 are implicated in MS susceptibility, it is important to note that the phenotypic differences observed in our study cannot be explained by the presence of HLA-A2 and HLA-A3 because MS patients and control subjects were matched by these alleles.

Due to the stringent criteria employed for myelin epitope validation, this study focused on a relatively limited number of myelin CD8^+^ T cell determinants. It is therefore possible that other myelin CD8^+^ T cell epitopes may exist and show unique differences in frequency in MS patients. While myelin-specific CD4^+^ T cells have been directly linked to MS pathogenesis (18, 56), a similar role for myelin-specific CD8^+^ T cells remains unknown and is outside the scope of the present study. Although many studies support a proinflammatory role of myelin-specific CD8^+^ T cells in MS and EAE, it remains possible that they may have a regulatory or suppressive function in certain circumstances (57–59). It is also important to acknowledge that the antigen specificity of clonally expanded T cells in MS is not known at this time, and it is possible that other self- or even non–self-antigens may be targeted (59). Indeed, it was recently reported that CNS-infiltrating CD4^+^ T cell clones recognize ubiquitously expressed self-antigens (56, 60).

Deciphering which CD8^+^ T cells are relevant to the disease process and the unique characteristics they possess is a critical hurdle to our understanding of MS. In this study, a pMHC I tetramer-based approach was used to characterize multiple myelin-specific CD8^+^ T cell populations ex vivo, which demonstrated evidence of increased activation in MS patients. This unique activation state of myelin-specific CD8^+^ T cells was associated with their preferential depletion by anti-CD20 mAb therapy. These data provide further evidence that myelin-specific CD8^+^ T cells play a central role in MS pathogenesis. Selective targeting of these activated myelin-specific CD8^+^ T cells could represent an attractive approach to MS treatment.

## Materials and Methods

### Human Subjects and Samples.

All investigations were institutional review board approved (University of California, San Francisco, Committee on Human Research), and all participants provided written informed consent. Peripheral blood was collected from healthy controls (no known history of neurologic disease, immunocompromised state, or immunosuppression) and RR-MS patients as defined by 2017 McDonald Criteria. Ninety-two percent of RR-MS patients were naive to DMT, and the remaining patients had been off DMTs greater than 3 mo at the time of baseline sample collection (Table S1). For patients experiencing recent relapses, blood samples were collected after more than 30 d of last steroid treatment. One hundred to 140 mL of whole blood were collected from each subject. PBMCs were isolated by Ficoll separation and were frozen in 10% DMSO and 90% FBS in liquid nitrogen until the day of experimentation. Subjects were screened for HLA-A2 and HLA-A3 status by serotyping using the antibody clones BB7.2 (BioLegend) and GAP.A3 (eBioscience), respectively. Genetic confirmation was done by haplotyping of the MHC I A locus at 4× resolution (Histogenetics). PBMCs from 11 untreated HLA-A2^+^ and/or HLA-A3^+^ RR-MS patients were used in the initial epitope screening experiments. A second cohort of RR-MS (*n* = 24) and healthy donors (*n* = 17) matched for HLA-A2^+^ and HLA-A3^+^ status was used for ex vivo tetramer enrichment and phenotyping. A subset of the RR-MS patients (*n* = 9) were subsequently treated with anti-CD20 mAb (rituximab or ocrelizumab) and underwent a second blood collection 1 to 6 mo after their most recent infusion.

### Antigens.

Candidate myelin epitopes were selected from the myelin proteins MBP, PLP, MOG, MAG, and CNP. Using a combination of the epitope prediction databases IEDB and SYFPEITHI, 31 and 24 candidate myelin peptides were identified and synthesized to >95% purity (Genemed Synthesis) for HLA-A2 and HLA-A3, respectively (*SI Appendix*, Table S1). The influenza peptides M1_58–66_ (HLA-A2) and NP_265–273_ (HLA-A3) were used as control non-self-antigens. Myelin peptide:MHC relative binding of HLA-A2– and HLA-A3–expressing cell lines, T2-A2 (ATCC) and T2-A2/A3 (coexpressing HLA-A2 and HLA-A3; courtesy of Dr. Elizabeth Jaffee, Johns Hopkins, Baltimore, MD), was measured based on a previously published protocol (61). In brief, 1 × 10^5^ T2 cells were washed and resuspended in serum-free RPMI and pulsed overnight with 10 µg/mL of the indicated myelin peptide or no peptide in an incubator at 37 °C. The influenza peptides M1_58–66_ and NP_265–273_ were used as positive control reference peptides for HLA-A2 and HLA-A3, respectively. T2 cells were washed and stained with HLA-A2 FITC (BB7.2) or HLA-A3 APC (GAP.A3) and washed again before collection by flow cytometry (LSRFortessa). The surface expression of HLA-A2 and HLA-A3 was measured by mean fluorescent intensity (MFI). The percent relative binding was calculated as [(MFI_myelin_ _peptide_ − MFI_no_ _peptide_)/(MFI_reference_ _peptide_ − MFI_no_ _peptide_)] × 100, as shown in *SI Appendix*, Fig. S1.

### Tetramer Generation and Staining.

Ultraviolet (UV) photolabile pMHC I monomers for HLA-A2 and HLA-A3 were obtained from the NIH Tetramer Core. All pMHC I tetramers were generated by UV-ligand exchange as previously described (62). Tetramerization was carried out using streptavidin conjugated to fluorophores PE (Life Technologies), APC (Life Technologies), BV421 (BioLegend), or PE-CF94 (BioLegend). For all combinatorial tetramer staining experiments (see below), 500 µM d-biotin was added to each tetramer to quench any possible unloaded biotin-binding sites on streptavidin to prevent unintended binding of excess pMHC complexes to the incorrect fluorophore (33). All T cell samples were CD8 purified to greater than 90% purity by negative selection (human CD8^+^ T cell isolation kit; Miltenyi) prior to tetramer staining. CD8^+^ T cells were treated with 100 nM dasatinib (StemCell) for 30 min at 37 °C followed by staining (no wash step) with the indicated tetramers (final concentration, 2 to 3 µg/mL) for 30 min at room temperature. Tetramers were pooled together just prior to tetramer staining. Pairs of cognate antigen tetramers in dual fluorophores were used for all tetramer staining in order to improve binding specificity. Tetramers were used within 2 mo of synthesis for epitope screening experiments, and within 1 wk for ex vivo tetramer enrichment experiments. Cells were washed in FACS buffer (1× DPBS without calcium or magnesium, 0.1% sodium azide, 2 mM EDTA, 1% FBS) or sort buffer (1× DPBS without calcium or magnesium, 2 mM EDTA, 1% FBS, 0.22 µm sterile filtered), and then stained with anti-CD8 PECy7 (eBioscience; SK1) or Alexa 700 (BioLegend; SK1), a PerCP/Cy5.5 dump antibody mixture containing anti-CD4 (BioLegend; RPA-T4), anti-CD14 (BioLegend; HCD14), anti-CD16 (BioLegend; B73.1), and anti-CD19 (BioLegend; HIB19), and Aqua506 viability dye (Life Technologies), washed, and collected by flow cytometry (LSRFortessa). For the ex vivo tetramer enrichment experiments, anti–CD45RA-APC/Fire750 (BioLegend; HI100), anti-CCR7 PECy7 (BioLegend; G043H7), and anti-CD20 FITC (BioLegend; 2H7) were also used. CD8^+^ T cells were identified by lymphocytes, single cells (FSC-H, FSC-W), dump channel negative, live CD8^+^ cells (*SI Appendix*, Fig. S3*A*).

### Expansion of Myelin-Specific CD8^+^ T Cells.

PBMCs (6 × 10^6^ to 2.4 × 10^7^) from untreated MS patients were stimulated with pools of 5 myelin peptides (final concentration 10 µg/mL each) with recombinant human IL-2 (10 ng/mL) in 96-well plates (5 × 10^5^ cells/well) and incubated (37 °C, 5% CO_2_) for 1 wk. All T cell culture was done using RPMI 1640 with l-glutamine containing 10% heat-inactivated FBS (Life Technologies), 55 nM 2-mercaptoethanol (Life Technologies), penicillin/streptomycin/glutamine (Life Technologies), 1 mM sodium pyruvate (Life Technologies), MEM nonessential amino acids (Life Technologies), and MEM vitamin solution (Life Technologies). Peptides tested were those indicated in *SI Appendix*, Table S1. Wells from individual patient samples were pooled together, CD8 purified, treated with dasatinib, stained with pools of tetramers (containing the same cognate antigens from the primary stimulation), followed by surface staining as detailed above. Tetramer-positive CD8^+^ T cells were sorted on a FACSAria cell sorter into a 96-well round-bottom plate (50 to 100 cells per well). Sorted cells were stimulated for 2 wk with 2 µg/mL PHA-L (Sigma-Aldrich), 10 ng/mL recombinant human IL-2 (BioLegend), and irradiated (3,000 rad) allogenic feeder PBMCs. Cells were refed with half volume of fresh media and IL-2 every 3 to 4 d.

### Intracellular Cytokine Stimulation.

T2 cells (1 × 10^5^ cells per condition) expressing HLA-A3 and/or HLA-A2 were pulsed overnight with 10 µg/mL of the indicated myelin peptide or no antigen. Myelin tetramer-positive CD8^+^ T cells (5 × 10^4^ to 2 × 10^5^) were added to the pulsed T2 cells (total volume, 100 µL) and stimulated for 6 h in the presence of 1:500 GolgiStop (BD), 1:500 GolgiPlug (BD), and 1:200 CD28/CD49d (FastImmune; BD). Cells were washed with FACS buffer and stained with the cell surface antibodies as above (anti-CD8, dump channel antibody mixture, and live/dead dye). Cells were washed, fixed, and stained with antibodies for intracellular cytokines in permeabilization buffer. Intracellular antibodies included IFN-γ Alexa 647 (BioLegend; 4S.B3), TNFα Alexa 488 (BioLegend; Mab11), IL-2 BV421 (BioLegend; MQ1-17H12), GM-CSF PE (BioLegend; BVD2-21C11), CD107a APC/Cy7. Cells were then washed and collected on an LSRFortessa.

### Combinatorial Tetramer Staining and Enrichment.

In total, 3–5 × 10^7^ PBMCs from each human subject were thawed, washed, and CD8 purified by negative selection. The frequencies of antigen-specific CD8^+^ T cells were calculated as previously described (31, 63). After resuspending the purified CD8^+^ T cells in a precise volume of sort buffer (typically 100 to 120 µL), 5 µL was removed for counting with 123count eBeads (Invitrogen) in order to determine the total input number of CD8^+^ T cells (MS mean, 1.5 × 10^6^ CD8^+^ T cells; control mean, 1.9 × 10^6^ CD8^+^ T cells). An additional 5 µL was used for cell surface staining with anti-CD8, dump channel antibodies, CD45RA, CCR7, CD20, and viability dye. The remaining cells were used for tetramer enrichment and treated for 30 min with 100 nM dasatinib as above. CD8^+^ T cells from each human sample were stained with pools of HLA-A2– or HLA-A3–restricted myelin and influenza-specific tetramers. Staining for each antigen-specific tetramer was performed using dual fluorophores (*SI Appendix*, Fig. S2), where at least one of the fluorophores contained PE in order to allow enrichment with anti-PE magnetic beads. Tetramer-stained CD8^+^ T cells were washed, resuspended in 200 µL of sort buffer, and incubated with 50 µL of anti-PE magnetic microbeads (Miltenyi) for 20 min at 4 °C. Cells were washed and enriched over LS columns (Miltenyi) as per the manufacturer’s instructions. The tetramer-enriched cells were resuspended in a precise volume of sort buffer (typically 100 to 120 µL), and 5 µL was removed for counting with 123count beads in order to determine the total number of antigen-positive CD8^+^ T cells. The remaining cells were stained with same cell surface staining mixture as used for the preenriched samples. In addition to the gating strategy described above, a stringent tetramer gating strategy was employed (*SI Appendix*, Fig. S3), as previously described (33). In brief, tetramer-positive CD8^+^ T cells were gated on the dual fluorophores specified for each antigen (*SI Appendix*, Table S2). Only those tetramer-positive CD8^+^ T cells that were negative for the other fluorophores were considered genuine tetramer-positive cells. Thus, cells that stained positive with more or less than 2 fluorophores were excluded from the analysis.

### Statistics.

Statistical analyses and graphing were performed with GraphPad Prism, version 8. A 2-way ANOVA with multiple comparisons (corrected for false-discovery rate with 2-stage step-up method of Benjamini, Krieger, and Yekutieli) was used for comparisons between MS patients and control subjects. A mixed-effects model for repeated measures with multiple comparisons (corrected for false-discovery rate with 2-stage step-up method of Benjamini, Krieger, and Yekutieli) was used for comparisons of untreated and anti-CD20 mAb-treated samples. Values of *P* ≤ 0.05 were considered statistically significant.

### Data Availability.

All data and protocols used to support the findings of this study have been included in the manuscript and *SI Appendix*.

## Supplementary Material

Supplementary File
