# Supplementary material for: Anti-CD20 therapy depletes activated myelin-specific CD8+ T cells in multiple sclerosis
Source: Proc Natl Acad Sci U S A. 2019 Nov 20;116(51):25800–7. doi: 10.1073/pnas.1915309116 (PMC6926057; doi:10.1073/pnas.1915309116)
Supplement: Supplementary File [file pnas.1915309116.sapp.pdf]

PNAS
www.pnas.org

Supplementary Information for

Anti-CD20 therapy depletes activated myelin-specific CD8+ T cells in multiple sclerosis

Joseph J. Sabatino, Jr., MD/PhD, Michael R. Wilson, MD, Peter A. Calabresi, MD, Stephen L. Hauser, MD, Jonathan P. Schneck, MD/PhD, Scott S. Zamvil, MD/PhD

Corresponding authors
Joseph J. Sabatino, Jr., M.D., Ph.D.
Email: jsabatino@ucsf.neuroimmunol.org

Scott S. Zamvil, M.D., Ph.D.
Email: zamvil@ucsf.neuroimmunol.org

This PDF file includes:

Figures S1 to S6
Tables S1 to S2

www.pnas.org/cgi/doi/10.1073/pnas.1915309116

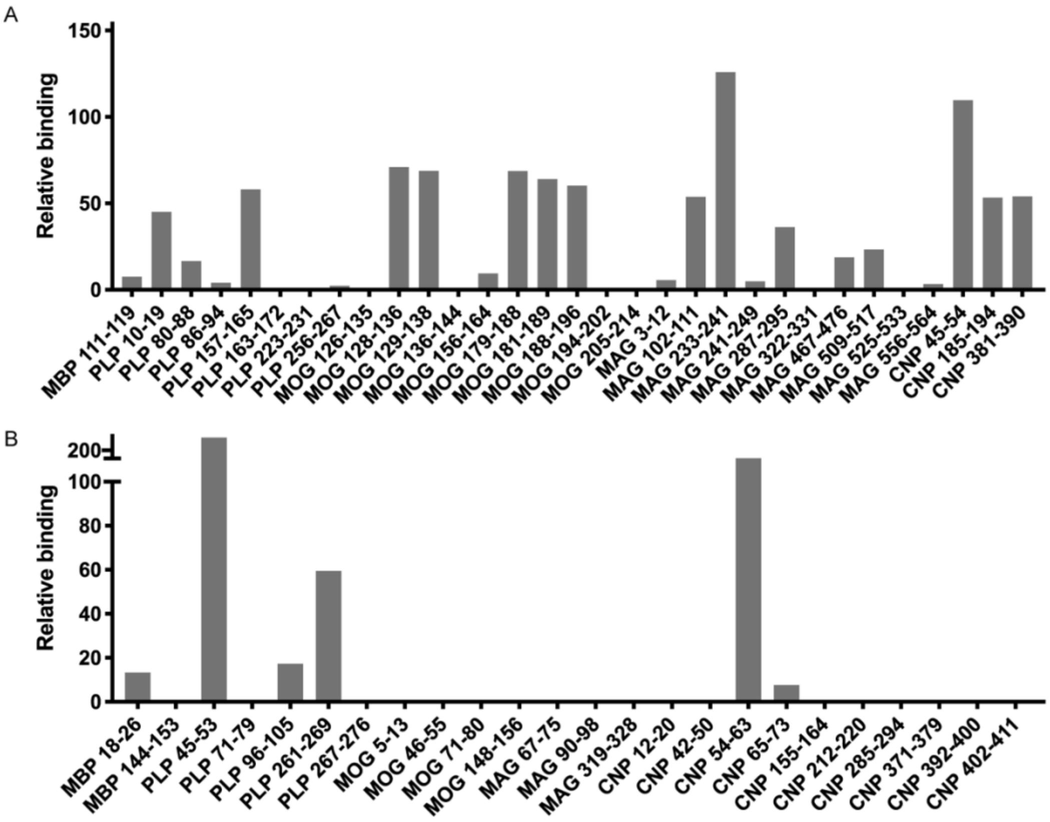

Fig. S1. The relative binding of different myelin peptides determined for T2 cells expressing HLA-A2 (A) and HLA-A3 (B) as described in Methods. Results are representative of two independent experiments for each HLA.

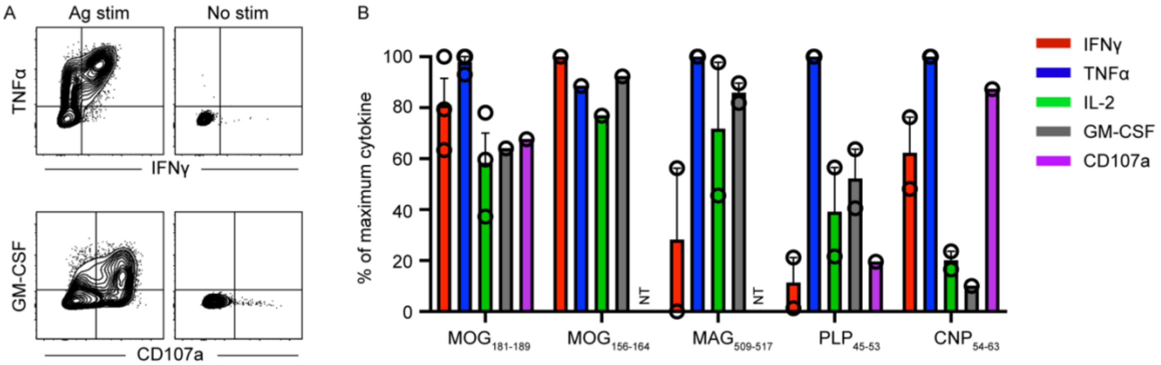

Fig. S2. Representative intracellular cytokine production of $PLP_{45\text{-}53}$:HLA-A3 $CD8^{+}$ T cells stimulated with or without cognate antigen (A). The relative percent cytokine production (normalized to percent of maximum cytokine produced) for the five validated myelin epitopes (B). Results are based on one to four patients for each epitope. CD107a was not tested (NT) for two of the indicated epitopes.

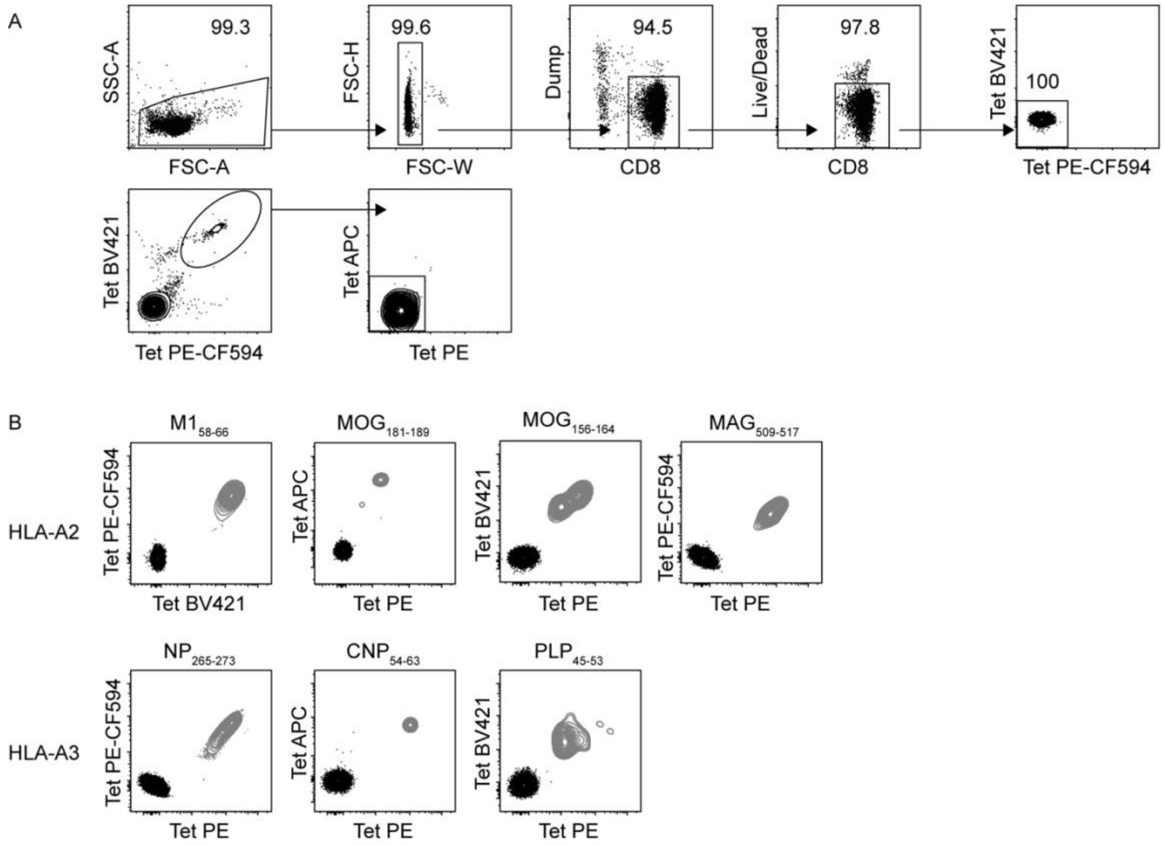

Fig. S3. The gating strategy for identifying tetramer-positive CD8+ T cells is shown. CD8+ T cells were identified by live single cell lymphocytes that were dump antibody cocktail negative (CD4/CD14/CD16/CD19) as shown in the top row of panel A. In pre-enrichment samples (i.e. tetramer unstained), no background signal was found in the channels used by the tetramers in post-enrichment. For post-enrichment samples (bottom row, panel A), CD8+ T cells were first gated on the two fluorophores specific for a particular antigen (see Supplemental Table 2). Double tetramer-positive cells were then gated on cells negative for the remaining non-specific fluorophores. Exclusion of cells binding to more or less than two fluorophores allows precise identification of antigen-specific double tetramer-positive CD8+ T cells (grey cells, panel B), which are shown relative to the tetramer-unstained pre-enrichment samples (black cells). Examples of enriched tetramer-positive CD8+ T cells is shown for each of the HLA-A2- and HLA-A3-restricted epitopes used in the study (panel B).

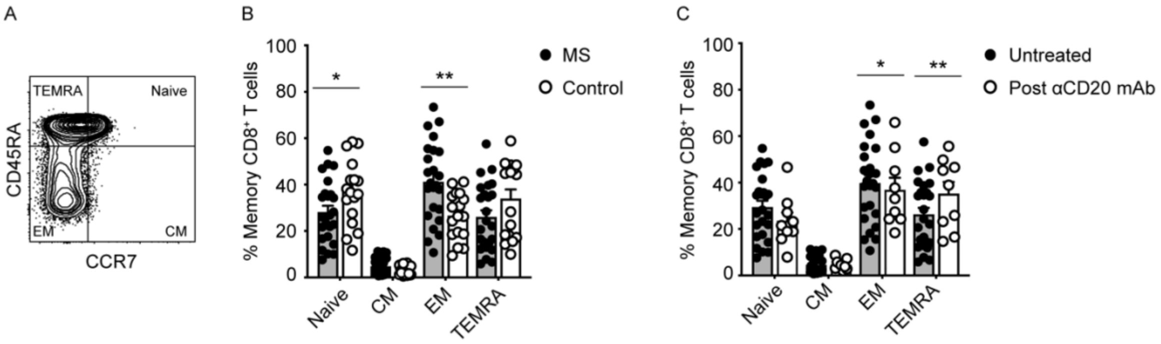

Fig. S4. Representative memory marker analysis used to identify naïve ($CCR7^+$ $CD45RA^+$), central memory (CM, $CCR7^+$ $CD45RA^-$), effector memory (EM, $CCR7^-$ $CD45RA^-$), or terminally differentiated effector (TEMRA, $CCR7^-$ $CD45RA^+$) $CD8^+$ T cell subsets (A). The mean percentages (± SEM) for each of the memory subsets in total unenriched $CD8^+$ T cells was compared between MS patients (n = 24) and control subjects (n = 17) (B) and between MS patients before (n = 24) and after anti-CD20 mAb treatment (n = 9) (C). A two-way ANOVA with multiple comparisons was used for comparisons between MS patients and control subjects and a mixed effects model for repeated measures with multiple comparisons was used for comparisons of untreated and anti-CD20 mAb treated samples (*p<0.05, **p≤0.001).

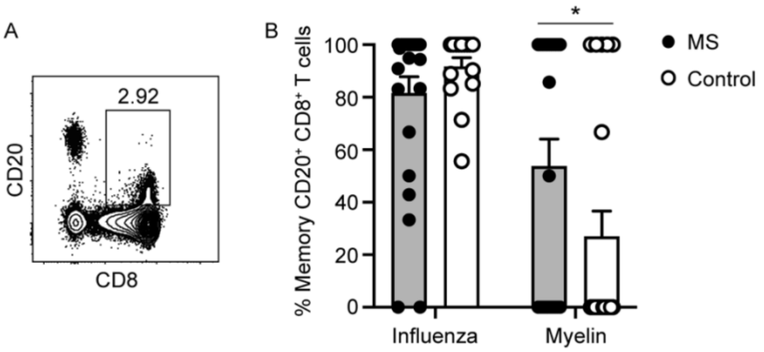

Fig. S5. Representative gating of CD20+ CD8+ T cells showing their identification by their “dim” CD20 expression pattern, which is distinct from CD20+ CD8- B cells (A). The percentage of memory CD20+ CD8+ T cells was compared between MS patients (n = 26 samples) and control subjects (n = 19 samples) for pooled influenza- and myelin-specific CD8+ T cells (B). Only samples with CD20+ T cell populations were included for analysis. A two-way ANOVA with multiple comparisons was used for comparisons between MS patients and control subjects (*p<0.05)

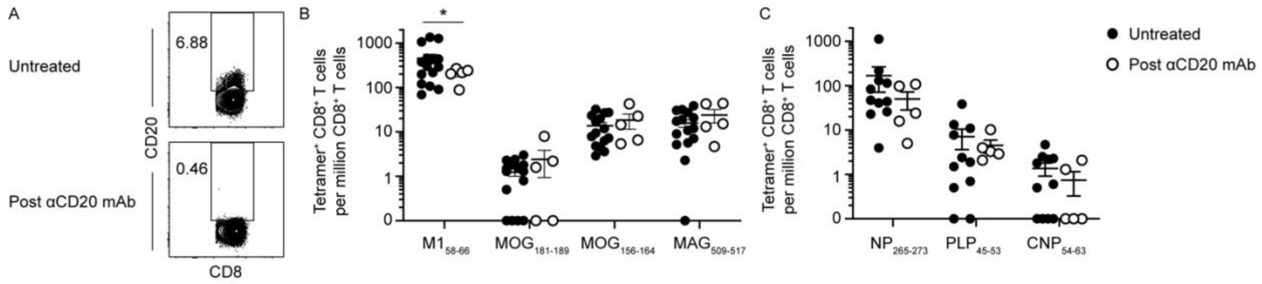

Fig. S6. CD20 expression of CD8+ T cells from a representative RR-MS patient before (top) and after anti-CD20 mAb treatment (bottom) is shown (A). The *ex vivo* frequencies of HLA-A2-restricted (B) and HLA-A3-restricted (C) of myelin- and influenza-specific CD8+ T cells is shown in RR-MS patients before (HLA-A2 n = 15, HLA-A3 n = 11) and after anti-CD20 mAb treatment (HLA-A2 n = 5, HLA-A3 n = 5). A mixed effects model for repeated measures with multiple comparisons was used for comparisons of untreated and anti-CD20 mAb treated samples (*$p<0.05$).

Table S1. Myelin peptides used in epitope screening.

HLA-A2		HLA-A3	
**Peptide**	**Sequence**	**Peptide**	**Sequence**
$MBP_{111-119}$	SLSRFSWGA	MBP_{18-26}	TASTMDHAR
PLP_{10-19}	CLVGAPFASL	$MBP_{144-153}$	GVDAQGTLSK
PLP_{80-88}	FLYGALLLA	PLP_{45-53}	KLIETYFSK
PLP_{86-94}	LLAEGFYTT	PLP_{71-79}	VIYGTASFF
$PLP_{157-165}$	YALTVVWLL	PLP_{96-105}	AVRQIFGDYK
$PLP_{163-172}$	WLLVFACSAV	$PLP_{261-269}$	ATYNFAVLK
$PLP_{223-231}$	NLLSICKTA	$PLP_{267-276}$	VLKLMGRGTK
$PLP_{258-267}$	MIAATYNFAV	MOG_{5-13}	VIGPRHPIR
$MOG_{126-135}$	VLVLLAVLPV	MOG_{46-55}	RVVHLYRNGK
$MOG_{128-136}$	VLLAVLPVL	MOG_{71-80}	LLKDAIGEGK
$MOG_{129-138}$	LLAVLPVLL	$MOG_{148-156}$	CLQYRLRGK
$MOG_{136-144}$	LLLQITVGL	MAG_{67-75}	KNYPPVVFK
$MOG_{156-164}$	KLRAEIENL	MAG_{90-98}	RLLGDLGLR
$MOG_{179-188}$	KITLFVIVPV	$MAG_{319-328}$	GLSVMYAPWK
$MOG_{181-189}$	TLFVIVPVL	CNP_{12-20}	FLPKIFFRK
$MOG_{188-196}$	VLGPLVALI	CNP_{42-50}	TVATLLECK
$MOG_{194-202}$	ALIICYNWL	CNP_{54-63}	ILRGLPGSGK
$MOG_{205-214}$	RLAGQFLEEL	CNP_{65-73}	TLARVIVDK
MAG_{3-12}	FLTALPLFWI	$CNP_{155-164}$	RLDCAQLKEK
$MAG_{102-111}$	LLLSNVSPEL	$CNP_{212-220}$	ELGNHKAFK
$MAG_{233-241}$	SMDVKYPPV	$CNP_{285-294}$	TISALFVTPK
$MAG_{241-249}$	VIVEMNSSV	$CNP_{371-379}$	KLYSLGNGR
$MAG_{287-295}$	SLLLELEEV	$CNP_{392-400}$	AIFTGYYGK
$MAG_{322-331}$	VMYAPWKPTV	$CNP_{402-411}$	KPVPTQGSRK
$MAG_{467-476}$	GLVLTSILTL		
$MAG_{509-517}$	LMWAKIGPV		
$MAG_{525-533}$	ILIAIVCYI		
$MAG_{556-564}$	VLFSSDFRI		
CNP_{45-54}	TLLECKTLFI		
$CNP_{185-194}$	FLPLYFGWFL		
$CNP_{381-390}$	MLTLAKNMEV		

The listed peptides were synthesized after *in silico* epitope prediction of the myelin proteins proteolipid protein (PLP), myelin basic protein (MBP), myelin oligodendrocyte glycoprotein (MOG), myelin associated glycoprotein (MAG), and 2',3'-cyclic-nucleotide 3'-phosphodiesterase (CNP) to the HLA-A2 and HLA-A3 alleles. After determining the best MHC I binders in a pMHC relative binding assay, the peptides highlighted in grey were tested for their ability to expand myelin-specific $CD8^{+}$ T cells from a cohort of untreated RR-MS patients.

Table S2. Tetramer staining scheme demonstrating the fluorophore pairs used for *ex vivo* $CD8^+$ T cell tetramer staining and enrichment for each antigen.

**HLA-A2**		**HLA-A3**	
**Antigen**	**Fluorophore pair**	**Antigen**	**Fluorophore pair**
$M1_{58-66}$	PECF594 + BV421	$NP_{265-273}$	PE + PE-CF594
$MOG_{181-189}$	PE + APC	CNP_{54-63}	PE + APC
$MOG_{156-164}$	PE + BV421	PLP_{45-53}	PE + BV421
$MAG_{509-517}$	PE + PECF594		
